# A duplex fluorescent quantitative PCR assay to distinguish the genotype I and II strains of African swine fever virus in Chinese epidemic strains

**DOI:** 10.3389/fvets.2022.998874

**Published:** 2022-09-23

**Authors:** Shinuo Cao, Huipeng Lu, Zhi Wu, Shanyuan Zhu

**Affiliations:** Jiangsu Key Laboratory for High-Tech Research and Development of Veterinary Biopharmaceuticals, Jiangsu Agri-animal Husbandry Vocational College, Taizhou, China

**Keywords:** diagnosis, fluorescent quantitative PCR, TaqMan probe, African swine fever virus genotype I, African swine fever virus genotype II

## Abstract

African swine fever (ASF) is a highly contagious hemorrhagic disease that affects domestic and wild pigs. A recent study reported that both ASF virus (ASFV) genotypes I and II have invaded farm-raised pigs in China, causing chronic infection and morbidity. To develop a duplex fluorescent quantitative PCR method to distinguish the ASFV genotypes I and II in Chinese epidemic strains, the probes and primers were designed based on the B646L sequences of genotypes I and II listed in the GenBank database. After optimizing the system, a duplex fluorescent quantitative PCR method for simultaneous detection of ASFV genotypes I and II B646L genes was successfully established. This method had no cross-reaction with Porcine circovirus type 2 (PCV2), Pseudorabies virus (PRV), or Porcine Parvovirus (PPV), indicating that it has strong specificity. The sensitivity results indicated that the minimum detection limit of ASFV genotypes I and II B646L was 10 copies/Rxn. The inter- and intra-group coefficients of variation were both <3%, indicating that the method was highly reproducible. Therefore, the established duplex fluorescent quantitative PCR assay is important for the differential detection and epidemiological investigation of ASFV.

## Introduction

African swine fever (ASF) is a fatal hemorrhagic swine disease that is highly contagious in all ages of pigs with high morbidity and mortality (1, 2). ASF virus (ASFV) is an enveloped, icosahedral, structurally complex, double-stranded DNA virus (3, 4). The disease is listed as a notifiable terrestrial and aquatic animal disease by the World Organization for Animal Health (OIE). The highly virulent ASFV isolates induce a peracute and acute disease characterized by hemorrhages in the skin and internal organs, loss of appetite and high fever. The mortality rate among pigs of all ages can be as high as 100%, and high mortality is an indicator of ASF. A less virulent strain produces mild clinical signs, such as slight fever, reduced appetite, and depression, that are often mistaken for other conditions in pigs and may lead to an incorrect diagnosis of ASF. Non-hemadsorbing avirulent strains cause subclinical non-hemorrhagic infections and seroconversion, although some animals may develop discrete lesions in the lungs or in the skin near bone-to-skin contact (5). The clinical course of ASF is typically acute or subacute. Subacute or chronic manifestations are more likely when less virulent strains are involved.

Since 1921, African swine fever has been endemic in many countries in Sub-Saharan Africa, particularly in Kenya. Consequently, it threatens food security and the livelihoods of poor and marginalized communities that raise domestic pigs as a means of subsistence (6–8). From Georgia in 2007, ASF spread to the Caucasus region and then to the European continent following its introduction in 2007 (9). A pig farm in Shenyang city in Liaoning Province reported the first ASF outbreak in mainland China in August 2018 (10). The virus has since rapidly spread to 32 provinces, autonomous regions, and municipalities, resulting in heavy losses for the pig industry and other related industries. In addition to the prevalence and direct economic losses from ASF, there have also been significant economic and social impacts, including restrictions on the transportation of pigs and pork products, restrictions on the slaughtering trade, insufficient supply of pork products, rising unemployment, and restrictions on global trade (11). There is no effective vaccine or treatment for controlling the spread of the disease in China at the moment.

According to the 3′-end sequences of the B646L gene that encodes p72, various genotypes of ASFV can be distinguished (12, 13). In Africa, at least 24 genotypes of ASF are naturally circulating. It is believed that all cases of ASFV spreading into Asia and Europe since 2007 are a result of a single epidemic of genotype II (14–16). An acute onset of infection caused by genotype I ASFV was first detected in Portugal outside Africa in 1957 (17). In China, the first ASFV isolates were grouped with genotype II based on sequence analysis (11, 18). China reported the emergence of genotype I ASFV and genotype II less virulent ASFV in 2021. Two strains, ASFV HeN/ZZ-P1/21 and SD/DY-I/21, with no porcine erythrocyte adsorption activity, were isolated from clinically infected pigs from Shandong and Henan farms (19). Genome-wide evolutionary analysis showed that these two strains were highly similar to the low-lethal genotype I strains NH/P68 and OURT88/3 isolated from Portugal in the last century. Both strains were quite different from the virulent genotype I strains L60 and Benin 97 isolated from Europe and Africa. It is generally recognized that ASF is a contagious and lethal disease, but if chronic infections spread, disease control will become more difficult.

Field identification of ASFV has become more challenging due to the emergence of genotype I ASFV in China. Although the fatality rate of this virus is low, it can cause chronic infection and morbidity in pigs. The virus has strong horizontal transmission ability in pigs. We developed a duplex fluorescent quantitative PCR assay for the differential detection of ASFV genotype I and II strains in this study. The method uses two different fluorescence signals derived from two differently labeled probes to differentiate ASFV genotype I and II strains simultaneously. As this assay has a short processing time, is relatively inexpensive and highly accurate. This approach described above should be generalizable and useful for dealing with these issues.

## Materials and methods

### Primers and probes

**Figure 1:**
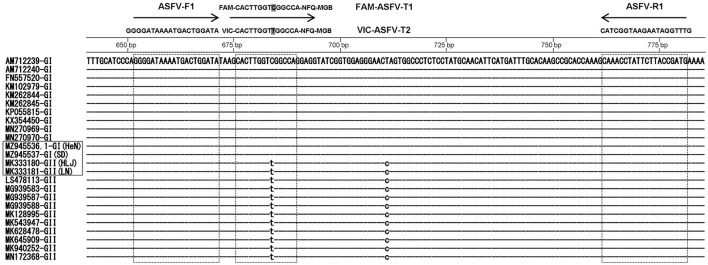
Nucleotide sequence alignment of B646L genes of African swine fever virus (ASFV) genotypes I and II with indicated position primers and TaqMan probes: ASFV-F1 primer, ASFV-S1 primer, FAM-ASFV-T1 probe and VIC-ASFV-T2 probe.

Beacon Designer 7.9 software was applied to design gene-specific probes and primers according to the B646L sequence of the P72 protein coding genes of ASFV Pig/HLJ/2018 (MK333180), DB/LN/2018 (MK333181), SD/DY-I/21 (MZ945537), and HeN/ZZ-P1/21 (MZ945536) strains published in GenBank. The primers ASFV-F3 (5′-GGG GAT AAA ATG ACT GGA TA-3′) and ASFV-R3 (5′-CAT CGG TAA GAA TAG GTT TG-3′) can be used for detection of both genotype I and II strains in Chinese epidemic strains. Based on an alignment of all ASFV strains, the primers were designed based on the conserved B646L regions of the genome. Sequence alignment was performed on the National Center for Biotechnology Information (NCBI) website (http://www.ncbi.nlm.nih.gov/BLAST/) (Figure 1). TaqMan probes, FAM-ASFV-T1-3 (FAM-5′-CACCTCCCTGCAGTCCCA-3′-NFQ-MGB) and VIC-ASFV-T2-3 (VIC-5′-CAC TTG GTT GGC CA-3′-NFQ-MGB), were designed to differentiate the genotype I strains from genotype II strains. The primers and probe sequences above were identified as ASFV-specific sequences by BLAST analysis. All probes and primers were synthesized by Tsingke Biotechnology Co., Ltd (Beijing, China).

### Viral genomic DNA extraction

Briefly, the genomic DNA was extracted from pseudorabies live vaccine (Bartha-K61 strain), porcine parvovirus disease live vaccine (WH-1), and inactivated porcine circovirus type 2 vaccine (LG) using a viral DNA extraction kit (Omega, Norcross, GA, USA). The DNA was eluted in an equal volume of elution buffer.

### Positive standards

The standard plasmids pUC57-ASFV-B646L-T1 and pUC57-ASFV-B646L-T2 were synthesized and sequenced by Tsingke Biotechnology Co., Ltd. Sequencing comparison indicated that the plasmid sequence was completely consistent with the B646L gene sequences of Pig/HLJ/2018 (MK333180) and SD/DY-I/21 (MZ945537) published in GenBank. Concentration of each DNA sample was measured using a spectrophotometer (Nanodrop-2000, Thermo Fisher Scientific, Waltham, MA, USA), and the number of copies was calculated. The plasmid was serially diluted 10-fold from 1 × 10^8^ to 1 × 10^2^ copies/Rxn and stored until use at −30 °C.

### Specificity of primers

Specificity of primers was detected by common PCR methods, and the prepared primers were used to identify the synthesized plasmid pUC57-ASFV-B646L. The reaction system volume was 20 μL: 0.25 μL LA Taq enzyme, 10 μL 2 × GC Buffer, 2 μL dNTP, 0.5 μL upstream and downstream primers, 1 μL plasmid, and ddH_2_O added for a total volume of 20 μL. The electrophoresis results were observed and photographed under a UV transilluminator.

### Optimization of the duplex fluorescent quantitative PCR

The equal volumes of both FAM-ASFV-T1 and VIC-ASFV-T2 were mixed in the same PCR tube containing pUC57-ASFV-B646L-T1 and pUC57-ASFV-B646L-T2 plasmids. The dual multiplex fluorescent quantitative PCR was performed by QuantStudio™ 3 (Thermo Fisher Scientific, Waltham, MA, USA). A set of primer and probe concentrations was optimized according to the final fluorescent signal values. The dual multiplex fluorescent quantitative PCR was performed as follows: 20 μL reaction mixture contained 0.5 μL of FAM-ASFV-T1 and VIC-ASFV-T2 (10 μM), 1 μL of ASFV-F1 and ASFV-R1 (10 μM), 2 μL of DNA, and 10 μL of Premix Ex Taq Probe qPCR (TaKaRa, Shiga, Japan). Annealing temperatures were set to 58, 59, 60, 61, 62, and 63°C *via* a temperature gradient. In this study, the amplification curve's cycle threshold (CT) value was defined as the number of cycles at which the curve reaches its threshold. Samples were considered positive if the CT value remained above the cutoff.

### The sensitivity, specificity and reproducibility of this assay

The following tests were conducted under optimized experimental conditions. This set of primers, probe and the plasmids pUC57-ASFV-B646L-T1, pUC57-ASFV-B646L-T2, or three non-ASFVs (PCV2, PPV, and PRV) were tested by the dual fluorescent quantitative PCR for evaluating the specificity of the assay. In addition, analytical sensitivity of the dual fluorescent quantitative PCR assay was tested using serially diluted positive standards. In addition, the copy number was taken into account when determining sensitivity. The repeatability (intra-assay) and reproducibility (inter-assay) of the dual fluorescent quantitative PCR assay was tested using the positive standards. We performed several batches of these experiments on different days, with three biological replicates per batch.

### Comparison of the duplex fluorescent quantitative PCR assay and ASFV UPL PCR

The standard plasmids were used as templates both in the real-time assay as described above and in an ASFV UPL PCR assay developed by Fernández-Pinero et al. (20). Comparing the two methods for detecting ASFV was carried out. Supplementary Table 2 summarizes the original sources and sequences of the primers and probes used in this study.

## Results

### Screening of primers and probes to distinguish ASFV genotype I and II strains by the duplex fluorescent quantitative PCR for Chinese epidemic strains

Based on FAM fluorescence signal (specific for ASFV genotype I) and VIC (specific for ASFV genotype II strain), three primer/probe sets were screened to distinguish ASFV genotype I and II strain by duplex fluorescent quantitative PCR for Chinese epidemic strains. The results revealed that the primer/probe sets 1 and 2 could not distinguish the ASFV genotype I and genotype II strains, while the primer/probe set 3 could detect and distinguish the two strains simultaneously, and with higher sensitivity (Table 1). Therefore, the primer/probe set 3 was used in subsequent experiments.

**Table 1:** Comparison of the efficiencies of the primer/probe sets of the duplex fluorescence quantitative PCR.

**Schemes**	**ASFV genotype I**				**ASFV genotype II**			
	**Probe 1**		**Probe 2**		**Probe 1**		**Probe 2**	
	**C_**T**_ value**	**Mean value**	**C_**T**_ value**	**Mean value**	**C_**T**_ value**	**Mean value**	**C_**T**_ value**	**Mean value**
Set 1	31.004	30.934	-	-	35.519	35.516	31.059	31.187
	30.809		-		35.421		31.534	
	30.989		-		35.607		30.968	
Set 2	30.985	30.961	-	-	44.577	43.548	32.127	32.092
	30.969		-		43.588		32.165	
	30.928		-		42.479		31.985	
Set 3	31.279	31.371	-	-	-	-	31.680	31.652
	31.290		-		-		31.427	
	31.545		-		-		31.850	

### The standard curves in the duplex fluorescent quantitative PCR assay

**Figure 2:**
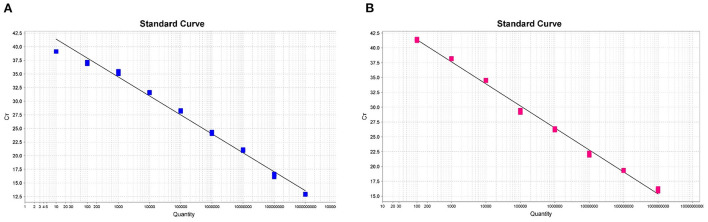
The standard curves of the dual fluorescent quantitative PCR for ASFV genotype I **(A)** and II **(B)** in Chinese epidemic strains. Based on the mean of Ct values against log10 of 10-fold serial dilutions of the synthesized plasmids pUC57-ASFV-B646L-T1 and pUC57-ASFV-B646L-T2.

In order to generate the duplex amplification curves and the fluorescent quantitative PCR standard curves, we used successively diluted known copy numbers of pUC57-ASFV-B646L-T1 and pUC57-ASFV-B646L-T2 for fluorescent quantitative PCR reaction under the optimized conditions. As shown in Figures 2A,B, the standard curves were linear in the range of 1 × 10^9^ to 1 × 10^1^ copies for the ASFV genotype I strain and in the range of 1 × 10^9^ to 1 × 10^2^ copies for the genotype II strain. The trendline for the genotype I strain showed a high degree of linearity (R^2^ = 0.989) (Figure 2A). The standard curve slope was −3.318, and the slope of intercept was 41.132. The regression formula for the standard curve was y = −3.318x + 41.132. The trendline for genotype II strain showed a high degree of linearity (R^2^ = 0.995) (Figure 2B). The standard curve slope was −3.713, and the slope of intercept was 48.787. The regression formula for the standard curve was y = −3.713x + 48.787. The duplex fluorescent quantitative PCR amplification efficiencies for the two targets were 100.160% (for genotype I) and 95.907% (for genotype II).

### Specificity of the duplex fluorescent quantitative PCR assay

**Figure 3:**
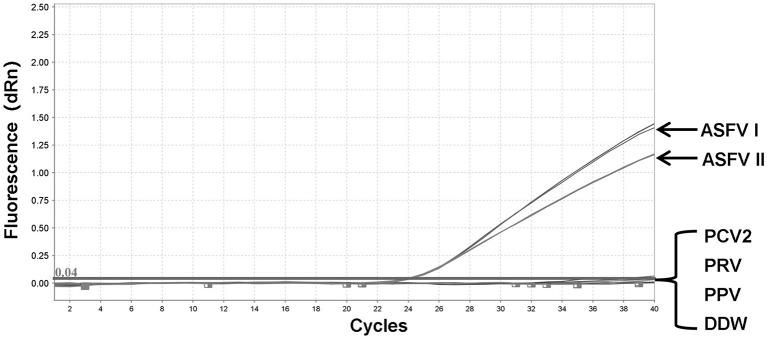
Specificity of the dual fluorescent quantitative PCR. No detection signal was obtained for Porcine circovirus type 2 (PCV2), Pseudorabies virus (PRV), or Porcine Parvovirus (PPV).

To validate the integrity of the extracted DNA, conventional PCRs were performed using the genes of three other porcine DNA viruses (PCV2, PRV, and PPV) (Supplementary Figure 1). The genomic DNA of PCV2, PRV, PPV, pUC57-ASFV-B646L-T1, and pUC57-ASFV-B646L-T2 plasmids was tested by fluorescent quantitative PCR assays. No specific signals were visible on the two channels for several non-ASFV porcine DNA viruses, including PCV2, PRV, and PPV, while the ASFV genotypes I and II could be distinguished correctly (Figure 3).

### Sensitivity of the duplex fluorescent quantitative PCR assay

The sensitivity of the assay was evaluated using the standard plasmids ranging from 1 × 10^9^ to 1 × 10^1^ copies/Rxn. In a fluorescent quantitative PCR assay, the 10-fold serial dilutions of the standard plasmids were simultaneously detected. Detection limits of the fluorescent quantitative PCR were 10 copies/Rxn and 100 copies/Rxn for ASFV genotype I and genotype II, respectively.

### Reproducibility of the duplex fluorescent quantitative PCR assay

Reproducibility of the assay was determined by examining whether ASFV genotypes I and genotype II could be distinguished between different inter- assay and intra-assay runs. The intra-assay and inter-assay variation of CT values was measured. As shown in Table 2, the developed method had good repeatability and stability. The intra-assay CV values of the fluorescent quantitative PCR ranged from 0.129 to 0.252% for ASFV genotype I and from 0.362 to 0.712% for ASFV genotype II. For comparison, ASFV UPL PCR was also performed on three concentrations of ASFV genotype I and II P72 positive plasmids used in the validation assay. The findings of this experiment for comparison of the duplex real-time PCR with ASFV UPL PCR are summarized in Supplementary Table 1.

**Table 2:** Repeatability evaluation of the duplex real-time PCR for intra-assay and inter-assay variation.

**Dilution (copies/Rxn)**	**P72 gene of ASFV genotype I**				**P72 gene of ASFV genotype II**			
	**intra-assay variation** ^**a**^		**inter-assay variation** ^**b**^		**intra-assay variation** ^**a**^		**inter-assay variation** ^**b**^	
	**(C_**T**_)^**c**^**	**CV (%)**	**(C_**T**_) ^**c**^**	**CV (%)**	**(C_**T**_) ^**c**^**	**CV (%)**	**(C_**T**_) ^**c**^**	**CV (%)**
10^4^	31.776 ± 0.080	0.252	32.157 ± 0.378	1.139	29.502 ± 0.210	0.712	28.624 ± 0.218	0.763
10^5^	28.736 ± 0.119	0.414	27.705 ± 0.753	2.717	26.150 ± 0.116	0.444	25.041 ± 0.673	2.687
10^6^	24.842 ± 0.032	0.129	22.694 ± 0.312	1.375	22.958 ± 0.083	0.362	20.967 ± 0.456	2.175

^a^Intra-assay variation was detemined on three repicates of recombinant plasmid dilutions analyzed in the same PCR run.

^b^Inter-assay variation was calculated on values obtained in three separate PCR runs.

^c^Mean ± standard deviation (SD).

CV coefficient of variation.

## Discussion

Since 2018, the first occurrence of ASF in China and a number of other countries has resulted in tremendous economic losses to the pig and related industries. A rapid and accurate diagnostic method is conducive to the early detection and control of ASFV. Recent studies have demonstrated that genotype I ASFV has invaded Chinese pigs, causing chronic infection and morbidity. Two ASFV isolates, HeN/ZZ-P1/21 and SD/DY-I/21, with no porcine erythrocyte adsorption activity were isolated from clinically infected pigs in Shandong and Henan provinces, respectively. Genome-wide evolutionary analysis showed that these two strains were highly similar to the low-lethal genotype I strains OURT88/3 and NH/P68 isolated from Portugal in the last century. They were quite different from the virulent genotype I strains L60 and Benin 97 isolated from Europe and Africa. Although the fatality rate from the genotype I strain is low, it can cause chronic infection and morbidity in pigs. The virus has strong horizontal transmission ability in pigs. Due to the slow course and various clinical manifestations caused by the infection, this strain results in more hidden prevalence, making early diagnosis difficult and presenting new challenges in the prevention and control of ASF. Therefore, effective countermeasures are needed as soon as possible.

Currently, there is no effective vaccine for ASFV in China, thus, the prevention and control of ASFV rely on monitoring and elimination of the virus. Among various established methods for the detection of ASFV, the HAD test can obtain accurate detection results within 24 h for high-potency ASFV, but it takes at least 6 days to determine a negative result. The test is often negative for low-virulence ASFV, and is thus unsuitable for rapid detection. ASFV antigen detection by ELISA is fast and accurate, but the detection sensitivity is low, because the low virus titer in subacute and chronically infected pigs, and false negative results tend to occur. TaqMan fluorescent quantitative PCR, as a specific, sensitive, and efficient detection method, has the advantage of early detection, thus facilitating early culling and reducing economic losses.

The molecular diagnostic techniques used in the diagnosis of ASF are conventional PCR assay (21) and fluorescent quantitative PCR assays (22), both of which are OIE recommended diagnostic methods. Although these methods have been widely used as two basic techniques to detect ASFV infections in the field, but they are not sensitive enough to observe only trace amounts of ASFV-positive samples. Also, they are not able to distinguish between the ASFV genotype I and II strain infections. In this report, a duplex fluorescent quantitative PCR assay was established based on B646L gene. This method can distinguish the ASFV genotypes I and II. It did not cross-react with PCV2, PRV, or PPV, and the assay had high specificity. The minimum number of tests for this method is 10 copies/Rxn. The TaqMAN-MGB probe based on the ASFV CP530R gene established by Wang Jianhua et al. and the fluorescent quantitative PCR based on ASFV B646L gene established by Zhang Quan et al. could detect at least 61 copies, and results with a minimum of 20 copies were with highly sensitive. The results are similar to those obtained using the K205R gene based on fluorescent quantitative PCR established by Guo Shaoping et al. that has a minimum detection volume of 10 copies and has high sensitivity. The coefficients of variation of repeatability tests between and within groups were all <0.712%. The results showed that the method had good repeatability. Therefore, the TaqMan fluorescent quantitative fluorescence quantitative method based on the ASFV p72 protein coding gene B646L established in this study is a specific, sensitive, and efficient detection method that can be applied in clinical practice.

## Conclusion

In summary, a new duplex fluorescent quantitative PCR method using TaqMan probe was developed, allowing simultaneous and differential detection of ASFV genotypes I and II with a rapid, sensitive, specific and reproducible capability. The developed method will contribute to the prevention and control of ASF in China.

## Data availability statement

The original contributions presented in the study are included in the article/Supplementary material, further inquiries can be directed to the corresponding author.

## Author contributions

SC and SZ conceived and designed the study. SC wrote the manuscript. SZ critically revised the manuscript. SC, HL, and ZU performed the majority of the experiments. All authors read and approved the final manuscript.

## Funding

This work was supported by a grant from the key project of Jiangsu Province's Key Reseach and Development plan (modern Agriculture) (BE2020407), the project of Jiangsu Agri-animal Husbandry Vocational College (NSF2022CB04, NSF2022CB25), and 2020 Taizhou Science and Technology Support Plan (Agriculture) Project (TN202001).

## Conflict of interest

The authors declare that the research was conducted in the absence of any commercial or financial relationships that could be construed as a potential conflict of interest.

## Publisher's note

All claims expressed in this article are solely those of the authors and do not necessarily represent those of their affiliated organizations, or those of the publisher, the editors and the reviewers. Any product that may be evaluated in this article, or claim that may be made by its manufacturer, is not guaranteed or endorsed by the publisher.
