# Supplementary figures and images for: A duplex fluorescent quantitative PCR assay to distinguish the genotype I and II strains of African swine fever virus in Chinese epidemic strains
Source: Front Vet Sci. 2022 Sep 23;9:998874. doi: 10.3389/fvets.2022.998874 (PMC9539676; doi:10.3389/fvets.2022.998874)

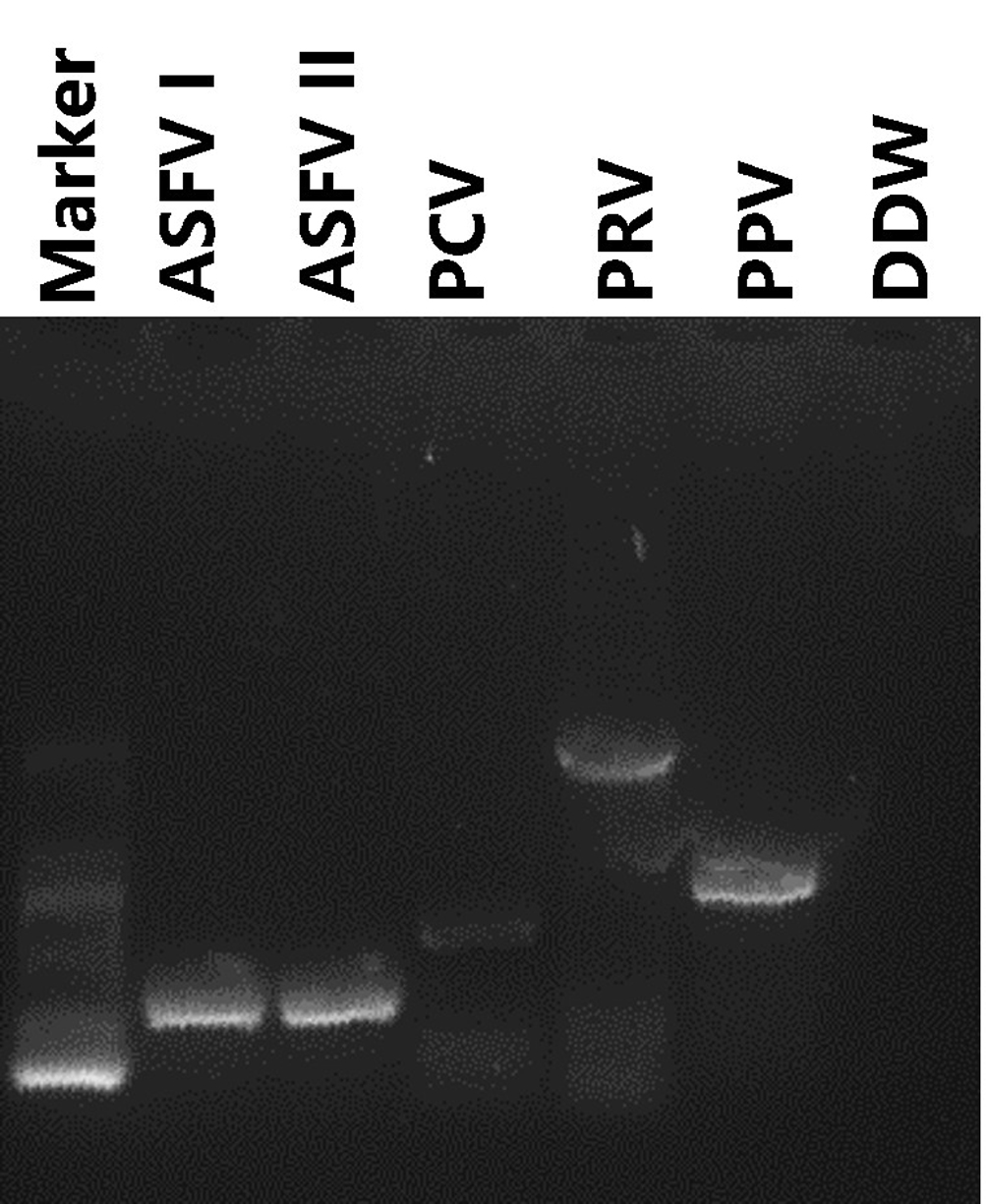

Supplement: Supplementary file 1 [file Image_1.JPEG]
