# Supplementary material for: A duplex fluorescent quantitative PCR assay to distinguish the genotype I and II strains of African swine fever virus in Chinese epidemic strains
Source: Front Vet Sci. 2022 Sep 23;9:998874. doi: 10.3389/fvets.2022.998874 (PMC9539676; doi:10.3389/fvets.2022.998874)
Supplement: Supplementary file 2 [file Table_1.docx]

Supplementary Table 1． Comparison of the duplex real-time PCR with ASFV UPL PCR

| Dilution (copies/Rxn) | P72 gene of ASFV genotype I | | | | P72 gene of ASFV genotype II | | | |
| --- | --- | --- | --- | --- | --- | --- | --- | --- |
|  | ASFV P72-I PCR | | ASFV UPL PCR | | ASFV P72-II PCR | | ASFV UPL PCR | |
|  | C_T_ value | Ct Mean | C_T_ value | Ct Mean | C_T_ value | Ct Mean | C_T_ value | Ct Mean |
| 10^3^ | 34.312 | 34.232 | 29.156  29.524  29.872 | 29.517 | 31.613 | 31.551 | 27.456  27.781  28.154 | 27.797 |
|  | 34.239 |  |  |  | 31.456 |  |  |  |
|  | 34.146 |  |  |  | 31.585 |  |  |  |
| 10^4^ | 31.340 | 31.530 | 26.230  26.037  26.922 | 26.396 | 27.896 | 28.329 | 25.124  25.482  26.782 | 25.796 |
|  | 31.223 |  |  |  | 28.325 |  |  |  |
|  | 32.026 |  |  |  | 28.765 |  |  |  |
| 10^5^ | 27.124 | 27.036 | 22.382  23.038  22.924 | 22.781 | 25.702 | 25.136 | 21.418  21.786  21.027 | 21.410 |
|  | 27.076 |  |  |  | 25.058 |  |  |  |
|  | 26.908 |  |  |  | 24.648 |  |  |  |
