# Supplementary material for: A duplex fluorescent quantitative PCR assay to distinguish the genotype I and II strains of African swine fever virus in Chinese epidemic strains
Source: Front Vet Sci. 2022 Sep 23;9:998874. doi: 10.3389/fvets.2022.998874 (PMC9539676; doi:10.3389/fvets.2022.998874)
Supplement: Supplementary file 3 [file Table_2.docx]

Supplementary Table 2. Primer and probe sets used in this study

| Names | Target gene | Sequences (5'-3') | Amplicon size (bp) | R/Q dyes |
| --- | --- | --- | --- | --- |
| ASFV-F1 | ASFV B646L | CAYCATATATTGGGTGCA | 119 | **…** |
| ASFV-R1 |  | GGTTGTCCCAGTCATATC |  | **…** |
| FAM-ASFV-T1-1 |  | FAM-CATTCGTCCTGGCA-NFQ-MGB | **…** | FAM/NFQ-MGB |
| VIC-ASFV-T2-1 |  | VIC-CATTCATCCTGGCA-NFQ-MGB | **…** | VIC/NFQ-MGB |
| ASFV-F2 | ASFV B646L | GCTTGTAGATCCYTTTG | 156 | **…** |
| ASFV-R2 |  | GTGACATCCGAACTATA |  | **…** |
| FAM-ASFV-T1-2 |  | FAM-AAGACCTATTGTACCCG-NFQ-MGB | **…** | FAM/NFQ-MGB |
| VIC-ASFV-T2-2 |  | VIC-AAGACCCATTGTACCCG-NFQ-MGB | **…** | VIC/NFQ-MGB |
| ASFV-F3 | ASFV B646L | GGGGATAAAATGACTGGATA | 130 | **…** |
| ASFV-R3 |  | CATCGGTAAGAATAGGTTTG |  | **…** |
| FAM-ASFV-T1-3 |  | FAM-CACTTGGTCGGCCA-NFQ-MGB | **…** | FAM/NFQ-MGB |
| VIC-ASFV-T2-3 |  | VIC-CACTTGGTTGGCCA-NFQ-MGB | **…** | VIC/NFQ-MGB |
| PCV2-F | PCV2 ORF2 | CTGTTTTCGAACGCAGTGCC | 466 | **…** |
| PCV2-R |  | CCCGCACCTTCGGATATA |  | **…** |
| PRV-F | PRV gB | CGGCATCGCCAACTTCTTC | 262 | **…** |
| PRV-R |  | GTCCTCCTTGAGCGTCTTCGT |  | **…** |
| PPV-F | PPV VP1 | ATACTTGGGGGAGGGCTT | 759 | **…** |
| PPV-R |  | GTTCCTGGGTGTTGGTCT |  | **…** |
| ASF-VP72-F |  | CCCAGGRGATAAAATGACTG | 67 |  |
| ASF-VP72-R | ASFV UPL PCR | CACTRGTTCCCTCCACCGATA |  |  |
| UPL#162 probe |  | 6FAM-GGCCAGGA-dark quencher dye |  | FAM/BHQ1 |
